# A Novel Technique of Breast Reconstruction: Inflation of Breast Tissue Expander with Air

**DOI:** 10.1097/GOX.0000000000002036

**Published:** 2018-12-14

**Authors:** Matthew Green, Habib Tafazal, Raghavan Vidya

**Affiliations:** From the Department of Breast Surgery, New Cross Hospital, Wednesfield Way, Wolverhampton, West Midlands, United Kingdom.

## BACKGROUND

Over the last decade, there has been an increase in implant-based immediate breast reconstruction, and single-stage breast reconstruction using fixed volume implants are carried out due to advances in biomaterials. However, 2-stage implant reconstruction still forms a significant proportion of breast reconstructions.^1^ Traditionally, tissue expanders (TEs) used in 2-stage reconstructions are inflated with saline: initially in theater and subsequently in clinic. We propose a novel method of inflation of TE with air and discuss its advantages over traditional methods.

## PROOF OF CONCEPT

**Fig. 1.**
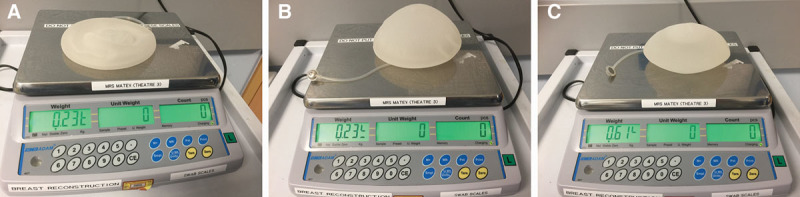
A, Demonstrating the dry weight of the implant. B, Demonstrating the weight remains unchanged while the volume expands after filling 400 cc of air. C, Demonstrates the increase in weight after filling with 400 ml of saline.

We postulate that inflation of air in TE expands the volume while the implant weight is almost unchanged after inflation of air. To demonstrate (Fig. 1A) this, we weighed the TE initially (236 g). We then inflated 400 cc of air (Fig. 1B), which expanded the implant volume while the weight remain unchanged (236 g). Subsequently, we injected 400 cc of saline, which clearly demonstrated volume expansion with increase in weight of the implant (Fig. 1C; 614 g). Hence, inflation of air achieves the desired volume expansion with minimal change in weight of the implant. Thus, air inflation would achieve the desired uniform volume expansion with least pressure on skin flaps and enhance wound healing.

We then tested our hypothesis in 5 patients (after obtaining informed consent) who underwent 2-stage implant-based breast reconstruction under a single surgeon (senior author). The desired size of TE was chosen for the immediate implant breast reconstruction, placed in the prepectoral plane, and the reconstruction was completed. Air inflation was carried out after the wound completely healed and after a minimum period of 6–8 weeks. The amount of air inflated depended on the degree to which the implant could be safely inflated without causing pressure on the skin flaps. Patient’s age ranged between 50 and 65 years and air insufflation ranged between 300 and 550 cc. We observed that the patients tolerated it very well and found the implant to be very light. We also found that the majority of air was retained following expansion and the air was retained until conversion to definitive implant 3–6 months later.

The use of air for the inflation of TEs has a number of advantages over the traditional saline expansion, exerting less pressure on the mastectomy skin flaps while maintaining TE volume, thus likely resulting in less venous congestion of skin flaps in the immediate postoperative setting. Additionally, air distributes equally through the expander at lower volumes, unlike saline, thus giving more uniform expansion early in the process.

Patients found it to be convenient and the breasts were reported to be lighter and comfortable. The use of air for inflation may pose a theoretical risk of further auto-inflation and TE may rupture when flying; hence it needs to be deflated. Devices using carbon dioxide inflation have recently been described.^2^

Thus, the novel technique of air to inflate TEs provides an exciting alternative to traditional expansion technique and further large studies are needed.
